# Real Life Data on Efficacy and Safety of Azacitidine Therapy for Myelodysplastic Syndrome, Chronic Myelomonocytic Leukemia and Acute Myeloid Leukemia

**DOI:** 10.1007/s12253-018-00574-0

**Published:** 2019-01-06

**Authors:** Grzegorz Helbig, Karolina Chromik, Krzysztof Woźniczka, Anna J. Kopińska, Kinga Boral, Martyna Dworaczek, Anna Koclęga, Anna Armatys, Marta Panz-Klapuch, Mirosław Markiewicz

**Affiliations:** School of Medicine in Katowice, Department of Hematology and Bone Marrow Transplantation, Medical University of Silesia, 40-032, Dąbrowski street, 25 Katowice, Poland

**Keywords:** Azacitidine, Myelodysplastic syndrome, Chronic myelomonocytic leukemia, Acute myeloid leukemia, Results

## Abstract

The administration of azacitidine (AZA) was found to be more effective than conventional care regimen (CCR) in patients with higher-risk myelodysplastic syndromes (MDS), chronic myelomonocytic leukemia (CMML) and acute myeloid leukemia (AML) with lower blast count. We designed a study to determine efficacy and safety of AZA therapy in “real life” patients with MDS, CMML and AML. The study included 83 patients (65% male) with a median age at diagnosis of 68 years. 43 patients were diagnosed with higher-risk MDS, 30 had AML and 10-CMML. Median AZA dose was comparable between treated groups. AZA dose reduction was required for 44% of MDS, 17% of AML and 25% of CMML patients. Complete remission (CR) was achieved in 14% of MDS, 7% of AML and 10% of CMML patients. Overall response rate was following: 27% for MDS, 20% for AML and 20% for CMML. Estimated OS at 12 months was 75% for MDS, 60% for AML and 75% for CMML. Median follow-up for MDS/AML/CMML from AZA initiation to last follow-up was 9.0, 9.4 and 9.4 months, respectively. The most common toxicity of AZA therapy was myelosuppression and infections. AZA treatment was effective in a limited number of patients with acceptable safety profile.

## Introduction

The unique mechanism of action of azacitidine (AZA) is associated with inhibition of DNA methyltransferase, enzyme which is thought to be responsible for DNA methylation. As a consequence, gene expression is altered which leads to reactivation of epigenetically silenced suppressor genes [1].

AZA is a hypomethylating agent known to be effective for the treatment of higher-risk myelodysplastic syndrome (MDS) and acute myeloid leukemia (AML) with lower blast count. The treatment protocol including AZA at a dose of 75 mg/m^2^ per day for 7 days every 28 days significantly increased overall survival (OS) when compared with conventional care regimen (CCR) [2]. There were also some data suggesting that AZA increased OS in older, newly diagnosed AML patients with >30% blasts with acceptable safety profile [3]. Finally, AZA treatment resulted in complete response in about 20% of patients with chronic myelomonocytic leukemia (CMML), especially in those without blasts in peripheral blood and low CMML-specific prognostic scoring system (CPSS) [4]. Discontinuation of AZA treatment while patients remained in partial or complete remissions led to rapid loss of response with a median of progression-free survival of 4 months [5].

AZA therapy was generally well-tolerated and the most common grade 3/4 adverse event was myelosuppression. Slower recovery of blood parameters was responsible for treatment delay or dose reduction. Toxicity of AZA was usually transient and decreased over time [6].

Herein we present our “real life” data on efficacy and safety of AZA therapy in MDS/AML/CMML patients treated in our center.

## Patients and Methods

Eighty three patients (43 with MDS, 30 with AML and 10 with CMML) received AZA at starting dose of 75 mg/m^2^ subcutaneous daily on days 1–7 in inpatient setting. Patients were recruited between 2012 and 2016 in our center in Katowice, Poland. IPSS was used to determine prognosis in MDS patients while CPSS estimated prognosis in those with CMML. Diagnosis of MDS/AML/CMML was established according to WHO classification [7]. Peripheral blood and trephine biopsy/aspirate were performed prior to AZA treatment. Cytogenetics testing was performed on all study patients prior to AZA initiation, however the results were obtained in 65% of subjects. Bone marrow aspirate was repeated after every third cycle of AZA unless there were features of disease progression in peripheral blood. Treatment response was defined according to standard response criteria [8]. Median age was comparable between MDS, AML and CMML patients (67 years vs 68 years vs 67 years; *p* = 0.37). Some younger patients (<60 years old) received AZA treatment as a patient’s choice or were found to be unfit for intensive chemotherapy or allogeneic stem cell transplantation. Female to male ratio did not differ between groups (0.4 vs 0.6 vs 0.4; *p* = 0.84). The similar proportion of patients among studied groups received prior therapy for their disorders; 35% in MDS (*n* = 15); 46% in AML (*n* = 14) and 40% in CMML (*n* = 4); *p* = 0.59. Pre-AZA treatment for MDS patients included low doses (LD) Ara-C ± steroids (*n* = 15). AML group had previously received LD Ara-C (*n* = 10) or 2 + 5 regimen (*n* = 4; daunorubicin and Ara-C). Before AZA administration, CMML patients were treated exclusively with hydroxyurea (*n* = 4). Five younger patients were proceeded to allogeneic stem cell transplantation from unrelated donor after achieving at least partial response to AZA (*n* = 3) or AML induction-like regimen (3 + 7 protocol; *n* = 2). Two of transplanted subjects remained alive at last contact. There was no difference in blood parameters at study entry between groups except for leukocyte count which was significantly increased in CMML when compared with MDS (9.6 × 10^9^/L vs 2.5 × 10^9^/L; *p* = 0.003) and AML (9.6 × 10^9^/L vs 2.2 × 10^9^/L; *p* = 0.003). Details were presented in Table 1. 56% of MDS/AML patients and 40% of CMML patients required red blood cells (RBCs) transfusions prior AZA initiation. Platelet support was required in 19% of MDS patients, 23% of AML and 30% of CMML.

**Table 1:** Patients’ characteristics at study entry

Characteristic	MDS (*n* = 43)	AML (*n* = 30)	CMML (*n* = 10)
Age; years, (median; range)	67 (41–84)	68 (43–82)	67 (54–82)
Sex; male/female	30/13	18/12	7/3
WBC (×10^9^/l); (median; range)	2.5 (0.8–11.6)	2.2 (0.9–9.8)	9.6 (2.5–35.8)
HGB (g/dl); (median; range)	9.5 (4.7–14.5)	9.5 (6.2–14.0)	9.7 (7.7–11.8)
PLT (×10^9^/l); (median; range)	67 (10–292)	72 (5–272)	57 (14–253)
Blasts cells in PB (%); (median; range)	11.2 (0–18)	0 (0–30)	1.5 (0–20)
Blasts cells in BM (%); (median; range)	9 (9–19)	22 (20–49)	13 (3–20)
Cytogenetic results (n; %)	31 (72)	15 (50)	8 (80)
complex karyotype	7	2	1
normal karyotype	17	10	7
monosomy 7	3	–	–
other	4	3	–
failed	12	15	2
RBCs transfusion-dependence	24	17	4
PLT transfusion-dependence	8	7	3
IPSS*		NA	NA
Intermediate risk II	25		
High risk	6		
CPSS*	NA	NA	
Low			1
Intermediate I			3
Intermediate II			4

AML = acute myeloid leukemia; CMML = chronic myelomonocytic leukemia; CPSS=CMML prognostic scoring scale; HGB = hemoglobin; IPSS = international prognostic scoring scale; MDS = myelodysplastic syndrome; PLT = platelets; RBC = red blood cells; WBC = white blood count; * = assessed only for the patients with available cytogenetic results

## Statistical Analysis

Nonparametric comparisons of group means were performed by using the Mann-Whitney *U* test. Proportions were compared by Fisher exact test. The distribution for overall survival (OS) was estimated using the method of Kaplan and Meier and compared using the log-rank test. A *p* value less than .05 was considered significant. All computations were performed with StatSoft Poland analysis software (version 10.0).

## Results

**Fig. 1:**
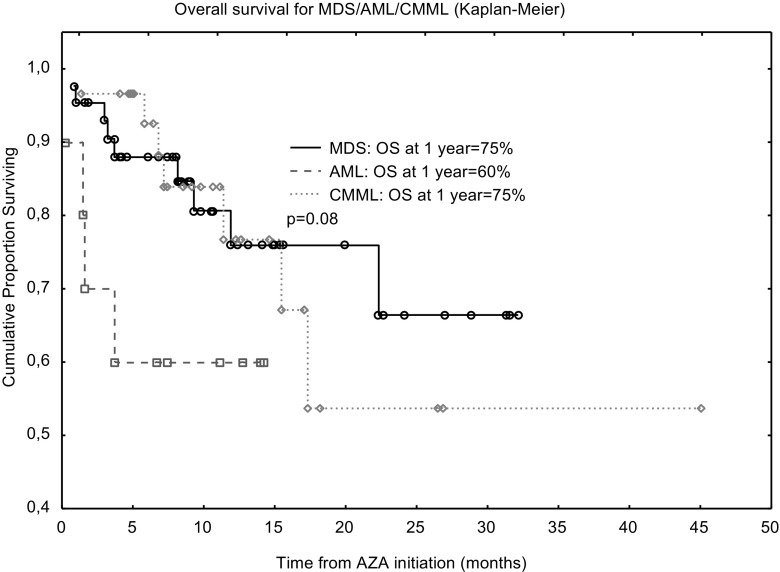
Overall survival curves for studied groups

Median AZA dose for single cycle was comparable between treated groups: 950 mg (range 150–1100) for MDS, 950 mg (700–1050) for AML and 925 mg (700–1050) for CMML. The median number of AZA cycles was 6 (range: 3–23) for MDS cohort whereas AML and CMML patients received a median of 4 AZA cycles (range 3–26 and 3–11, respectively). Median time from diagnosis to AZA commencement did not differ between groups and was following: 12.3 months (range 1–45.8), 10.9 months (range 1.4–99.3) and 9.3 months (range 0.3–30.5), for AML/MDS/CMML, respectively. AZA dose reduction was required for 44% of MDS, 17% of AML and 25% of CMML patients. No CMML patient required AZA cycle delay during the treatment whereas 13% of MDS/AML patients had their AZA dose postponed. Median follow-up for MDS/AML/CMML from AZA initiation to last follow-up was 9.0, 9.4 and 9.4 months, respectively. Median follow-up from diagnosis to last contact was 12.3, 10.9 and 9.4 months for MDS/AML/CMML respectively. Estimated OS at 12 months was 75% for MDS, 60% for AML and 75% for CMML (*p* = 0.08) see Fig. 1.

Complete remission (CR) was achieved in 14% of MDS, 7% of AML and 10% of CMML patients. Overall response rate (ORR) including CR, partial response (PR) and hematological improvement (HI) was as follows: 27% for MDS, 20% for AML and 20% for CMML. Most patients achieved their response after median of 3 AZA cycles, however there were 4 patients who met CR criteria after first AZA cycle. In responders, response duration varied from 3 months to >24 months while still on AZA. Stable disease was observed in 51% of MDS, 37% of AML and 40% of CMML patients. Disease progression (PD) was demonstrated for 21% of MDS, 43% of AML and 40% of CMML patients. Finally, the mortality rate for MDS, AML and CMML patients was 21%, 23% and 40%, respectively. Details are shown in Table 2.

**Table 2:** Response rates in azacitidine-treated patients

Response to azacitidine	MDS (*n* = 43;%)	AML (*n* = 30;%)	CMML (*n* = 10;%)
CR	6(14)	2(7)	1(10)
PR	3(7)	1(4)	0
HI	3(7)	3(9)	1(10)
SD	22(51)	11(37)	4(40)
PD	9(21)	13(43)	4(40)
Death rate	9(21)	7(23)	4(40)

CR = complete response; HI = hematological improvement; PR = partial response; PD = disease progression; SD = stable disease

More than 50% of treated patients in each group experienced no adverse events. The most common side effects of AZA treatment were allergic reactions at injection site, myelosuppression and infections. There have been 4 cases of severe pneumonia with the presence of *K. pneumoniae* in blood culture. One patient developed urinary tract infection and one- sinusitis. Three patients developed septic shock which resulted in death. A single case of AH1N1 influenza infection was observed. One patient died suddenly due to stroke. Details are shown in Table 3.

**Table 3:** Azacitidine treatment-related complications

Parameter	MDS (*n* = 43; %)	AML (*n* = 30; %)	CMML (*n* = 10; %)	Total * (*n* = 83;%)
No side effects	23 (53)	17 (56)	5 (50)	45 (54)
Myelosuppression de novo or its deterioration without infection	7 (16) G2	1 (3) G2	1 (10) G1	9 (11)
Myelosuppression-related infections	3 (7) G3	4 (13) G3–4	1 (10) G1	8 (10)
Infections without myelosuppression	2 (5) G4	5 (17) G3–4	1 (10) G4	8 (10)
Injection site reaction to AZA	10 (23) G1–2	8 (26) G1–2	3 (30) G1–2	21 (25)
Diarrhea	3 (7) G2	2 (7) G1–2	1 (10) G1	6 (7)
Creatinine increase	3 (7) G1–2	0 (0)	1 (10) G2	4 (5)
Transaminases increase	1 (3) G2	1 (3) G2	0	2 (2)
Stroke	1 (3) G4	0 (0)	0 (0)	1 (1)

*one patient may have developed more than one complication

G1–4 = side effects grading according to WHO

## Discussion

The efficacy of AZA therapy has been demonstrated for the first time for patients with high-risk MDS in 2 randomized phase III studies: AZA-001 and the Cancer and Leukemia Group B (CALBG) [2, 9]. AZA was administered at a dose of 75 mg/m^2^/day for 7 days of each 28-day cycle in both of those studies and its efficacy was compared with best supportive care (CALBG) or CCR (AZA-001). In the CALBG study, 60% of patients treated with AZA achieved response while in best supportive care (BSC) group response was observed in 5%. Of note is, that no patient achieved CR/PR in BSC group in contrast to 23% of patients in AZA group. AZA treatment when compared to BSC was associated with longer time to leukemic transformation (LT) and prolonged overall survival especially in those patients ≥65 years. 90% of patients treated with AZA achieved their response by sixth cycle [9, 10]. The efficacy of AZA treatment in MDS was later confirmed in the AZA-001 trial which compared this agent with CCR (conventional induction chemotherapy, low dose Ara-C, BSC). AZA if compared with CCR improved OS, increased the median time to LT and demonstrated significantly higher response rate with CR of 17% vs 8%; *p* = 0.01 [2].

Of note is, that presented results come from well-designed clinical trials with precisely defined eligibility criteria. It is obvious, that these results cannot be directly implemented to daily clinical practice where we face with the “unselected” group of patients with low performance status, co-morbidities, abnormal laboratory findings and who had frequently received prior chemotherapy.

Having those limitations in mind we wanted to determine, what is the efficacy and safety profile of AZA therapy in “real-life” population of patients with MDS/AML/CMML. This retrospective analysis was evaluated separately for each group of studied patients. CR rates were comparable between groups with the highest CR rate demonstrated for MDS (14%) and the lowest for AML (7%). Overall response rates were also comparable and fluctuated in the range of 20% for each study cohorts. Of note is, that our CR rates were in line with those obtained for patients who participated in 2 randomized phase III studies [2, 9]. The comparable CR rates were also demonstrated for “real-life” MDS/AML patients in a small study presented by Turkish Study Group [11]. In contrast, about 40% of MDS patients achieved CR in a study by Isabella et al. [12]. Interestingly, the authors observed neither CR nor PR in their AML/CMML patients. Unexpectedly high CR rate after AZA treatment was demonstrated for “real life” Belgian patients; 41% for MDS/CMML and 44% for AML with estimated 1-year OS of 57% [13].

There was a difference in ORRs between our results and those presented by others; ~20% in our cohort if compared with ~50% in the most cited studies [2, 9, 11, 12]. Of note is, that about 20% more patients achieved HI in the latter studies if compared with our data. This discrepancy in response rates may result from our relatively short median follow-up (~9 months) since responses may still occur beyond 9 months of AZA treatment [14].

Our study groups were comparable in terms of median AZA dose per cycle and median number of administered AZA cycles. The starting AZA schedule was unified for all cohorts, however a significant proportion of patients required AZA dose reduction or delay (57% with MDS, 20% with AML and 25% with CMML). These data did not differ from those reported by others [2, 10].

The median number of cycles required to achieve response was 3 and this was in line with that presented by other authors [2]. Of note is, that in a minority of patients (*n* = 4), a CR has already been achieved after the first AZA cycle. On the other hand, there have been also late responses suggesting that treatment continuation may be appropriate when safe.

Recently, the combination of AZA with BCL2 inhibitor- venetoclax has shown promising results in phase 1 study of elderly patients with untreated AML. Overall, 61% of study patients achieved complete or near complete remission. The regimen was well tolerated with neutropenia and thrombocytopenia remaining the most common grade 3–4 adverse events [15].

It should be highlighted, that our study has some important limitations. The three study subgroups (AML/MDS/CMML) were too small to consider each one as an independent series. Due to the same reason, the statistical evaluation was not performed for patients who received azacitidine as a first-line treatment and for those who were treated after prior therapies.

AZA therapy was well-tolerated with only transient toxicity decreasing over time [10]. About 50% of our patients experienced no toxicity effects of AZA administration. The side effects observed in our study were in line with those presented by other authors [2, 9]. The most common non-hematological adverse event was injection site reaction to AZA which presented itself as erythema. No patient developed pyrexia or bleeding complications. The new onset myelosuppression or its deterioration during therapy with concomitant infections were responsible for AZA dose delay or dose reduction in a significant proportion of patients. Infections were not common, however they were life-threatening and resulted in severe pneumonia followed by septic shock in 3 patients. Of note is, that infection-related mortality in patients on AZA affects 23% of patients, and occurs more frequently during the first 2 cycles of treatment [16].

## Conclusions

The efficacy of AZA therapy for “real life” patients with MDS/AML/CMML was modest and lower if compared with that from clinical trials. The minority of studied patients achieved complete remission and there were no differences in overall response rates between studied cohorts. The safety profile was acceptable, however one may be aware of severe life-threatening infectious complications. In summary, despite all above, we recommend this type of therapy in daily clinical practice because no other rational options exist to date.

## References

[1] Stresemann C, Lyko F (2008). Modes of action of the DNA methyltransferase inhibitors azacitidine and decitabine. Int J Cancer.

[2] Fenaux P, Mufti GJ, Hellstrom-Lindberg E, Santini V, Finelli C, Giagounidis A, Schoch R, Gattermann N, Sanz G, List A, Gore SD, Seymour JF, Bennett JM, Byrd J, Backstrom J, Zimmerman L, McKenzie D, Beach C, Silverman LR (2009). International Vidaza High-Risk MDS Survival Study Group. Efficacy of azacitidine compared with that of conventional care regimens in the treatment of higher-risk myelodysplastic syndromes: a randomized, open-label, phase III study. Lancet Oncol.

[3] Dombret H, Seymour JF, Butrym A, Wierzbowska A, Selleslag D, Jang JH, Kumar R, Cavenagh J, Schuh AC, Candoni A, Recher C, Sandhu I, Bernal del Castillo T, al-Ali HK, Martinelli G, Falantes J, Noppeney R, Stone RM, Minden MD, McIntyre H, Songer S, Lucy LM, Beach CL, Dohner H (2015). International phase 3 study of azacitidine vs conventional care regimens in older patients with newly diagnosed AML with >30% blasts. Blood.

[4] Alfonso A, Montalban-Bravo G, Takahashi K, Jabbour EJ, Kadia T, Ravandi F, Cortes J, Estrov Z, Borthakur G, Pemmaraju N, Konopleva M, Bueso-Ramos C, Pierce S, Kantarjian H, Garcia-Manero G (2017 Mar. 28) Natural history of chronic myelomonocytic leukemia treated with hypomethylating agents. Am J Hematol 92:599–606. 10.1002/ajh.24735 [epub ahead of print]. 10.1002/ajh.24735 PMC5553721 28370097

[5] Cabrero M, Jabbour E, Ravandi F, Bohannan Z, Pierce S, Kantarjian HM, Garcia-Manero G (2015). Discontinuation of hypomethylating agent therapy in patients with myelodysplastic syndromes or acute myelogenous leukemia in complete remission or partial response: retrospective analysis of survival after long-term follow-up. Leuk Res.

[6] Santini V, Fenaux P, Ghulam M (2010). Management and supportive care measures for adverse events in patients with myelodysplastic syndromes treated with azacitidine. Eur J Haematol.

[7] Vardiman JW, Thiele J, Arber DA, Brunning RD, Borowitz MJ, Porwit A, Harris NL, le Beau MM, Hellström-Lindberg E, Tefferi A, Bloomfield CD (2009). The 2008 revision of the WHO classification of myeloid neoplasms and acute leukemia, Rationale and important changes. Blood.

[8] Cheson BD, Greenberg PL, Bennet JM (2006). Clinical application and proposal for modification of the international working group (IWG) response criteria in myelodysplasia. Blood.

[9] Silverman L, Demakos E, Peterson B (2002). Randomized controlled trial of azacitidine in patients with the myelodysplastic syndrome: a study of the cancer and leukemia group B. J Clin Oncol.

[10] Navada SC, Silveraman LR (2017). Safety and efficacy of azacitidine in elderly patients with intermediate to high-risk myelodysplastic syndromes. Ther Adv Hematol.

[11] Ozbalak M, Cetiner M, Bekoz H, Atesoglu EB, Ar C, Salihoglu A, Tuzuner N, Ferhanoglu B (2012). Azacitidine has limited activity in “real life” patients with MDS and AML: a single Centre experience. Hematol Oncol.

[12] Isabella C, Avanzini P, Merli F. 5-Azacitidine in patients with myelodysplasia and acute myeloid leukemia: a single centre experience. Blood. (ASH Annual Meeting Abstracts) 2010; 116: Abstract 4969.

[13] Beguin Y, Selleslag D, Mers S (2015). Safety and efficacy of azacitidine in Belgian patients with high-risk myelodysplastic syndromes, acute myeloid leukaemia, or chronic myelomonocytic leukaemia: results of a real-life, non-interventional post-marketing survey. Acta Clin Belg.

[14] Voso MT, Niscola P, Piciocchi A (2015). Standard dose and prolonged administration of azacitidine are associated with improved efficacy in a real-world group of patients with myelodysplastic syndrome or low blast count acute myeloid leukemia. Eur J Haematol.

[15] DiNardo CD, Pratz KW, Letai A (2018). Safety and preliminary efficacy of venetoclax with decitabine or azacitidine in elderly patients with previously untreated acute myeloid leukaemia: a non-randomised, open-label, phase 1b study. Lancet Oncol.

[16] Trubiano JA, Dickinson M, Thursky KA, Spelman T, Seymour JF, Slavin MA, Worth LJ (2017). Incidence, etiology and timing of infections following azacitidine therapy for myelodysplastic syndromes. Leuk Lymphoma.

